# Supplementary material for: Prevalence and associated factors of adolescent fatherhood in Ethiopia: A multilevel analysis using the 2016 Ethiopian demographic health survey data
Source: PLoS One. 2021 Mar 29;16(3):e0249024. doi: 10.1371/journal.pone.0249024 (PMC8006980; doi:10.1371/journal.pone.0249024)
Supplement: Attachment — Submitted filename: Response to reviewer.docx [file pone.0249024.s001.docx]

**Author's point by point response to editor and reviewers comments**

**Title: Prevalence and associated factors of adolescent fatherhood in Ethiopia: a multilevel analysis using the 2016 Ethiopian demographic health survey data.**

**Manuscript number:** PONE-D-20-22844

**Subject: Submitting a revised version of the manuscript**

We would like to thank the reviewers and editor for sharing their view and novel scholarly experiences. The comments are very imperative which we strongly believe in improving the manuscript. We try to address all the comment raised by the revivers and academic editor line by line in the main document. The point-by-point responses for each of the comments, questions, and the revised manuscript are provided in the attached documents.

Thank you for considering our manuscript again.

**Response to editor**

1, Please ensure that your manuscript meets PLOS ONE's style requirements, including those for file naming.

**Authors response:** thank you editor: we have ensure that our manuscript was prepared based on PLoS one formatting style.

2, please complete your Competing Interests on the online submission form to state any Competing Interests. If you have no competing interests, please state "The authors have declared that no competing interests exist." as detailed online in our guide for authors at <http://journals.plos.org/plosone/s/submit-now>. This information should be included in your cover letter; we will change the online submission form on your behalf.

**Author’s response:** thank you editor: We have complete the information about competing interest in the online submission system and we have also included the competing interest information in the revised cover letter.

3, If there are ethical or legal restrictions on sharing a de-identified data set, please explain them in detail (e.g., data contain potentially identifying or sensitive patient information) and who has imposed them (e.g., an ethics committee). Please also provide contact information for a data access committee, ethics committee, or other institutional body to which data requests may be sent. If there are no restrictions, please upload the minimal anonymized data set necessary to replicate your study findings as either Supporting Information files or to a stable, public repository and provide us with the relevant URLs, DOIs, or accession numbers.

**Author’s response:** thank you for raising this important issue: we have used the Ethiopian demographic health survey (EDHS) data obtained from measure DHs program which is available online at www.measuredhs.com. So the data is freely available online and every one can access it from [www.measuredhs.com](http://www.measuredhs.com). We have also included the information about data availability in the revised cover letter.

4, your ethics statement must appear in the methods section of your manuscript. If your ethics statement is written in any section besides the Methods, please move it to the methods section and delete it from any other section. Please also ensure that your ethics statement is included in your manuscript, as the ethics section of your online submission will not be published alongside your manuscript.

**Author’s response:** thank you for raising this important issue: we have taken the comment and moved the ethical statement to the method section and we have deleted it in the other section of the revised manuscript.

5, Please ensure that you refer to Figure 1 in your text as, if accepted, production will need this reference to link the reader to the figure.

**Author’s response:** thank you for raising this important issue: we have cited figure 1 in the main document of the revised manuscript

6, we note you have included a table to which you do not refer in the text of your manuscript. Please ensure that you refer to Table 3 in your text; if accepted, production will need this reference to link the reader to the Table.

**Author’s response:** thank you for raising this important issue: we have referred to all tables in the main document of the revised manuscript.

**Response to reviewer**

**1.** This manuscript assessed the prevalence and associated factors of adolescent fatherhood in Ethiopia. My main concern is the method the authors used to perform variable selections. In addition, it'd be interesting to include age when the first child was born to further examine whether any of the factors are associated with an earlier age of adolescent fatherhood. Below are my detailed comments.

**Author’s response:** Thank you reviewer for the comments. As we stated in the method section of the manuscript, we select variables for the final model by using the cut of value p-value less than 0.2 since most of the time this variables have the potential to be significant predictors in the multivariable analysis. Besides, we also check whether the model improved while we include variables and if the model show improvement while including the variable by looking the LLR then we retain that variable in the model. As you know there are different methods of variable selection methods based on the nature of the data as well as the number of covariates. We assessed whether there was multi-collinnearity between independent variables using Variance Inflation Factor (VIF) and Tolerance by running pseudo linear regression analysis as VIF is dependent on coefficient of determination, and the mean VIF was less than 5. So, in this study there was no multi-collinnearity between independent variables, if there was multi-collinnearity we planned to use Lasso, ridge or Elastic net for variable selection, since the presence of multi-collinnearity results the regression estimates of the coefficient unstable and the standard errors of the coefficient widely inflated. But unfortunately there was no multi-collinnearity that is why we don’t use elastic net or lasso method of variable selection. Besides, the number of independent variables used in this study were few. Regarding the age at first child birth, here the aim of this study was to investigate the prevalence and associated factor of adolescent fatherhood. The study participants were adolescent men aged 15-24 years, and therefore all 15-24 aged men may not have child. If we incorporate the age of the respondent when the first child was born only those men who have child will be our study participant and the prevalence of adolescent fatherhood will be 100%. Therefore, our study participants are all men aged 15-24 years he may have child or not since the objective of our study was to assess the prevalence of adolescent fatherhood and its associated factors. Overall, the age of respondent at first child birth was missed for those men who didn’t have child and if we include this variable in the analysis those men who didn’t have child will be dropped in the analysis since STATA are doing complete case analysis.

2. Data source, line 3: please add the abbreviation "(EAs)" after "Enumeration Areas".

**Author’s response:** thank you for your important comment: we take the comment and we have added an abbreviation in the revised version of our manuscript

3. Data source, line 9: additional "," before "households".

**Author’s response:** thank you for your important comment: We have corrected it in the revised version of our manuscript.

4. Study variables, line 5: please include more details on the variables included: 1) how they were measured (e.g., how contraceptive knowledge was assessed), and 2) how these variables were coded (e.g., was age treated as continuous variable or categorical variable).

**Author’s response: thank you for raising this very important issue;** based on the comment raised by the reviewer we made the correction in the revised version of the manuscript. We have included the detailed information about the independent variables in table 1 of our revised manuscript. We have also categorized age in two categories as indicated in table 1 (see table 1 page 16 to18).

 5. Data management and analysis: the authors included variables with p<0.2 from the bivariable analysis in the multivariable analysis. However, this is not the state-of-the-art approach to perform variable selection. As this study is exploratory, the authors can use models such as elastic net or lasso models to perform variable selection, along with the use of cross-validation to avoid over fitting.

**Author’s response:** Thank you reviewer for raising essential comments. As you stated very well our study was exploratory model but not predictive model. Because our model is explanatory model when we think of the presence of multi-collinnearity between independent variables we have to take intense measures unlike the predictive models. First of all before we have used the cut of values that was p-value less than 0.2, we have assessed the presence of multi-collinnearity using VIF and tolerance by doing pseudo linear regression analysis since the presence of multi-collinnearity could make the coefficient unstable and inflates the standard error. The mean VIF was less than 5 and therefore there was no multi-collinnearity. Initially as you stated we planned to use elastic net or lasso method of variable selection depending on the nature of the correlated variables in order to make the correction of multi-collinnearity during analysis but there was no multi-collinnearity that is why we used p-value less than 0.2 to select variables for the multivariable multilevel analysis. Besides, we also assessed whether a model is improved or not in every step while we include one variable in the model based on LLR value. Secondly, as our source of data were EDHS, the number of variables collected to assess factors associated with adolescent fatherhood were limited and therefore we don’t need to reduce the number of covariates in the analysis and over fitting problem is not our issue. This all what we have considered while we have done the study. Dear reviewer, if it doesn’t convince you we are ready to use Lasso or elastic net approach of variable selection.
