# Supplementary material for: Prevalence and associated factors of adolescent fatherhood in Ethiopia: A multilevel analysis using the 2016 Ethiopian demographic health survey data
Source: PLoS One. 2021 Mar 29;16(3):e0249024. doi: 10.1371/journal.pone.0249024 (PMC8006980; doi:10.1371/journal.pone.0249024)
Supplement: Attachment — Submitted filename: response to reviewers.docx [file pone.0249024.s002.docx]

**Author's point by point response to editor and reviewers comments**

**Title:** Prevalence and associated factors of adolescent fatherhood in Ethiopia; A multilevel analysis of the 2016 Ethiopian Demographic and Health Survey data

**Manuscript number:** PONE-D-20-22844

**Subject: Submitting a revised version of the manuscript**

We would like to thank the reviewers and editors for sharing their views and novel experiences. The comments are very imperative which we strongly believe in improving the manuscript. We address all the comments raised by the revivers line by line in the main document. The point-by-point responses for each of the comments, questions, and the revised manuscript are provided in the attached documents.

Thank you for considering our manuscript again.

**Authors’ response to reviewer**

1. I still recommend them to perform variable selections using the state-of-the-art data-driven methods. The current approach is likely to have over-fitting, which can be addressed using cross-validation

**Authors’ response:** Thank you reviewer for the comments. As per your suggestion, we have used the LASSO methods for variable selection to select variables for the final model. After installing the LASSO in STATA software by using SSC install lasso, then we run the model that is the logit model with the potential predictors and several models with corresponding out of sample-R^2^, BIC and cross-validation mean square error were reported. Since the outcome variable is binary and follows binomial distribution the R ^2^ was pseudo-R ^2^. Therefore, we select a model that has the lowest CV mean prediction error, the largest pseudo-R ^2,^ and the smallest BIC values. This model is selected as the best-fitted model in the absence of occupational status (in this model the variable occupation is removed). Overall, we incorporate those variables selected by the lasso method. The variables selected by the lasso method such as knowledge towards contraceptive, number of unions, respondent age, age of respondent at first sex, wealth status, education status, religion, region, residence and sex of household head were included in the final multivariable multilevel binary logistic regression analysis.
